# Transcranial Direct Current Electric Stimulation Combined with Physical Exercise in Patients with Greater Trochanteric Pain Syndrome: Randomized Clinical Trial

**DOI:** 10.3390/medsci14020312

**Published:** 2026-06-12

**Authors:** Eunice Fragoso Martins, Nicole Lie Okumura, Vívian Santos Xavier Silva, Ana Luiza Meneses de Oliveira, Cezar Sabino Pereira da Silva, Ana Clara Dias Pereira, Jean Marcos de Souza

**Affiliations:** 1 School of Medical Sciences, Universidade Estadual de Campinas (UNICAMP), Campinas 13083-881, SP, Brazil; e208215@dac.unicamp.br (E.F.M.); n223293@dac.unicamp.br (N.L.O.); c168758@dac.unicamp.br (C.S.P.d.S.); 2 School of Physical Education, Universidade Estadual de Campinas (UNICAMP), Campinas 13083-851, SP, Brazil; v195476@dac.unicamp.br; 3 School of Medicine, Pontifical Catholic University of Campinas (PUC-Campinas), Campinas 13010-920, SP, Brazil; analuimoliveira@gmail.com (A.L.M.d.O.); anaclaradiaspereira2@hotmail.com (A.C.D.P.)

**Keywords:** gluteal tendinopathy, greater trochanter pain syndrome, motor cortex, rehabilitation, transcranial direct current stimulation

## Abstract

Background/Objectives: Transcranial direct current stimulation (tDCS) has been explored as a strategy for pain management, but no study has investigated its use in Greater Trochanteric Pain Syndrome (GTPS). This study evaluated the effects of the combination of resistance exercises (REs) with tDCS on pain, functionality, and quality of life in patients with GTPS. Methods: In this randomized, double-blind trial, adults with GTPS were allocated to receive tDCS with RE (intervention group, IG) or sham tDCS with RE (control group, CG). Supervised 20 min sessions occurred on four consecutive days. Anodal tDCS (2 mA) was applied over the primary motor cortex. The primary outcome was the VISA-G.BR score at day thirty. Secondary outcomes included pain, functionality, and quality of life at multiple time points, assessed by HAGOS, PQAS, McGill Pain Questionnaire, and SF-36. Results: Thirty patients were included. Both groups improved, but between-group differences were nonsignificant for the primary outcome (VISA-G.BR effect size, −0.16; 95% CI, −0.54 to 0.27; *p* = 0.460). Secondary outcomes followed a similar pattern. Conclusions: These findings reinforce the value of RE in GTPS while suggesting a limited role for short-term tDCS protocols. Future studies should investigate whether protocols involving a greater number of stimulation sessions may produce superior clinical effects.

## 1. Introduction

Greater Trochanteric Pain Syndrome (GTPS) is a complex and multifactorial motor syndrome characterized by pain in the region of the greater trochanter of the femur. This pain is frequently associated with tendinopathy of the gluteus medius and minimus muscles, in addition to trochanteric bursitis [1]. It is a common clinical condition that affects up to 15% of the population over 50 years of age [2] and may occur equally in athletes and sedentary individuals, being slightly more prevalent in women than in men [1].

Risk factors for GTPS include obesity, previous knee pathologies such as knee osteoarthritis, and low back pain [3], as well as kinetic factors such as excessive hip adduction, muscle weakness, and overuse or misuse of the gluteal muscles [1].

This complex combination can lead to the development of tendinopathies, partial tendon tears, or entheseal damage in these muscles [4], which contributes to pain and can trigger new alterations in movement, perpetuating the condition. Although trochanteric bursitis may be present in a portion of patients, the majority of contemporary authors believe, based on anatomopathological and radiological studies, that bursitis is more of a secondary process to tendinopathy than the primary cause of the disease itself [5].

Taking into consideration this biomechanical theory as the pathophysiological basis of GTPS, Grimaldi and collaborators suggest a mechanism for the genesis of the process. Their work proposes that weakness of the gluteal musculature could lead to hypertrophy of the iliotibial structures, which would compress the tendons of the gluteus medius and minimus, causing further muscle weakness and, consequently, tendinopathy [1].

Since the origin of the tendinopathy appears to lie, at least in part, in gluteal weakness, clinical trials have already evaluated the role of abductor strengthening exercises and have reported attractive results [6]. However, in clinical practice, many patients do not adhere to rehabilitation programs, as functional limitation and pain at the beginning of treatment protocols pose major difficulties. Thus, strategies to re-educate hip movement kinetics may be useful for the initial rehabilitation of patients with GTPS, paving the way for more effective resistance exercise interventions thereafter.

Transcranial direct current electrical stimulation (tDCS) is a non-invasive, low-cost, and relatively safe method of stimulating the central nervous system, allowing the facilitation or inhibition of neuronal depolarization in a known region of the cortex [7], which modulates spontaneous neuronal firing [8]. The technique has already been used and evaluated in systematic reviews, demonstrating potential benefits in various pain disorders [8], regardless of whether they are mediated by central mechanisms of pain sensitization [9] or by peripheral mechanisms of pain [10], but it has not yet been tested in GTPS. In addition, the incorporation of physical exercises during tDCS sessions is potentially capable of producing greater effects than neurostimulation alone [11].

Given that stimulation of the motor cortex appears to facilitate the learning of movement patterns [12], we hypothesized that movement re-education promoted by tDCS sessions accompanied by low-load and low-volume exercises could promote gains in functionality, pain control, and quality of life in patients with GTPS, considering the results obtained in other types of dysfunctions. The present study aimed to evaluate whether the combination of resistance exercise with tDCS in daily assisted sessions is capable of promoting improvement in pain, functionality, and quality of life in patients with GTPS when compared to exercise alone.

## 2. Materials and Methods

### 2.1. Protocol and Registration

This study was conducted in accordance with CONSORT guidelines [13] and was previously registered in the Registro Brasileiro de Ensaios Clínicos (REBEC) under the number RBR-95x5kmk (registration date: 20 January 2023). The study was conducted in accordance with the Declaration of Helsinki and received approval from the Ethics Committee of Universidade Estadual de Campinas (registration number 61187322.8.0000.5404, approval statement number 5.674.167). All participants provided written consent to participate in the study. Additionally, its protocol and feasibility phase were previously published [14], and all raw data related to the study are publicly available at: https://doi.org/10.25824/redu/RTEK2K (accessed on 9 June 2026).

### 2.2. Inclusion and Eligibility Criteria

Patients were recruited personally by the researchers at the outpatient clinics of Internal Medicine and Rheumatology of the tertiary teaching hospital (Hospital de Clínicas) of the Universidade Estadual de Campinas, São Paulo, Brazil. All patients waiting for consultation were questioned about hip pain and were subsequently invited to participate and evaluated by a certified physical therapist if they answered positively to a brief structured screening questionnaire (Supplementary File S1).

Inclusion and exclusion criteria were defined a priori. To meet inclusion criteria, participants of both sexes needed to be adults (≥18 years old) and have GTPS as defined by the criteria proposed by Grimaldi and collaborators [15]. These criteria define GTPS when palpation of the gluteus medius insertion at the greater trochanter elicits pain greater than 3 (on a scale of 0 to 10), associated with at least one of the following additional criteria:

1. Presence of trochanteric pain after remaining for 30 s in a single-leg stance;
2. Pain on the resisted isometric abduction maneuver;
3. Pain on the resisted flexion, adduction, and internal rotation maneuver.

Participants were excluded for the following reasons: motor or cardiovascular restrictions preventing physical training—even of low intensity; metal implants; decompensated psychiatric illness; poorly controlled epilepsy; history of seizures or blackouts; extensive skin lesions that would hinder the placement of tDCS electrodes; psoriasis; and pregnancy.

### 2.3. Experimental Design

Randomization was performed by an investigator not otherwise involved in recruitment, treatment, assessment, or data analysis (P.C.E.P.) using the allocation system provided by the stimulation device manufacturer (MicroEstim–NKL, Brusque, SC, Brazil). The neurostimulator operated through 200 preprogrammed, randomly generated five-digit codes corresponding to either active or sham tDCS. A decoding list linking each code to its stimulation mode was provided by the manufacturer and remained concealed from the investigators until completion of data collection.

Randomization sequences were generated in Excel (Microsoft Corporation, WA, USA) and stratified by sex and age group based on the epidemiology of GTPS [2,16]. Therefore, stratification was weighted approximately as follows: 70% of participants were aged 40–60 years, 10% were younger than 40 years, 20% were older than 60 years, and 75% were female.

Allocation concealment was ensured using opaque sealed envelopes identified only by stratum. Participants and investigators remained blinded throughout the study; however, no method was used to assess the success of the blinding strategy. At each session, the assigned code was entered into the neurostimulator, which automatically delivered either active or sham stimulation. The sham protocol was designed to closely mimic active stimulation, minimizing the likelihood of unblinding. Participants were randomized to: (1) receive tDCS associated simultaneously with low-volume and low-intensity resistance training focused on the hip and thigh musculature (Intervention Group–IG) or (2) receive sham tDCS associated with the same exercise protocol (Control Group–CG).

Participants were assessed using translated and validated scales immediately before the start of the protocol, right after the last session (4 days later), 30 days after, and 60 days after the intervention. The data analysis sought statistically relevant differences between the four time points of the protocol and baseline.

### 2.4. Assessment of Outcomes

To assess the clinical parameters of pain and functionality, participants answered the following questionnaires at the four previously described time points:

1. Victorian Institute of Sport Assessment–Gluteal Tendinopathy, Brazilian version (VISA) [17]: assesses pain, function, and activity in patients with gluteal tendinopathy.
2. Copenhagen Hip and Groin Outcome Score, Brazilian version (HAGOS) [18]: assesses pain, symptoms, function, physical/sports activities, and quality of life in patients with hip/groin disease.
3. Pain Quality Assessment Scale, Brazilian version (PQAS) [19]: assesses the patient’s perception of pain—intensity, sensations, and spatial/temporal characteristics.
4. McGill Pain Questionnaire (MPQ) [20]: assesses the subjective experience of pain in multiple dimensions.
5. Medical Outcomes Short-Form Health Survey-36 (SF-36) [21]: assesses health-related quality of life across eight physical and mental domains.

Safety and adherence data were gathered descriptively by the researchers during the training sessions. The adherence rate was calculated as the number of training sessions the participant effectively attended in relation to the total number of prescribed sessions. Adverse events, in turn, were reported and rated in accordance with the Common Terminology Criteria for Adverse Events (CTCAE) of the National Institutes of Health [22].

The primary endpoint of the study was a significant difference in VISA scores 30 days after the protocol between the IG and the CG. Secondary outcomes were pain, functionality, and quality of life, assessed using HAGOS, MPQ, PQAS, and SF-36 at the time points immediately after the intervention and 30 and 60 days later, both between groups and across time points (to assess the isolated effect of training). The VISA was also assessed at the other time points.

### 2.5. Intervention Protocol

The intervention was carried out over four consecutive days of physical training, assisted by medical professionals, physical therapists, or physical educators, at the Laboratório de Biomecânica e Reabilitação do Aparelho Locomotor (LABRAL) of the Hospital de Clínicas of the Universidade Estadual de Campinas (UNICAMP). The training consisted of the following exercises, adapted from the GLoBE protocol [23]: squat, single-leg stance, step-up, seated hip abduction, hip raise, and walking (a comprehensive explanation of the exercises with pictures is provided in the Supplementary File S2). The training lasted 20 min, and, during intervals, participants performed static marching for 30 s to maintain the stimulus—the complete description of the protocol was previously published [14].

tDCS was performed with the anode positioned over the primary motor cortex (C3 or C4), according to the dominant hemisphere. The cathode was positioned on the contralateral supraorbital region (Fp1 or Fp2). The regions selected were determined in accordance with the International 10–20 System [24] using cephalic surface measurements, according to the protocol.

The electrodes were applied with sponges moistened with saline solution and fixed with elastic bands. A current of 2 mA was administered for 20 min—the total duration of the protocol. The parameters of current intensity, application time, and time interval between applications were based on previous studies using tDCS [7,10,11]. The choice of only four sessions aimed to improve adherence as much as possible while maintaining the minimum number of days sufficient to produce neuroplasticity [12,25,26]. This number is also aligned with the results from our pilot study [14].

The device used was the MicroEstim from the brand NKL (Brusque, SC, Brazil), in which the rise of the current was progressive throughout the first minute of stimulation, allowing for the evaluation of participant tolerance. The CG was subjected to the same assembly of the device, following the manufacturer’s sham stimulation protocol.

In the sham protocol, the device performed the ramp-up phase normally until reaching the programmed current intensity. After 30 s, stimulation was automatically ramped down without any visual indication on the display, which continued to show a normal countdown timer, programmed current, and simulated impedance values.

The brief initial stimulation was designed to mimic the cutaneous sensations of active tDCS and maintain blinding. After ramp-down, the device emitted minimal monitoring pulses (6 ms, 200 μA) only to detect electrode disconnection. The resulting average current was considered physiologically insignificant and insufficient to produce neuromodulatory effects while still providing a highly convincing stimulus.

Throughout the study, neither the participants nor the investigators were aware of the allocation. Participants were instructed to maintain the medications they were using prior to enrollment.

### 2.6. Statistical Analysis

As described previously [14], according to the study that validated the VISA, the mean baseline value of patients with GTPS was 62.82 (±15.75) [17], and according to a study by Ebert and colleagues, the minimally important change in the VISA was twenty-nine points [27] on a scale of one hundred (therefore, 29%). Thus, admitting an α error of 5% and a β error of up to 20%, the sample size was calculated at 12 participants for each arm of the study. Accounting for dropouts, we prepared the study to recruit 30 volunteers.

The results are presented as mean ± standard deviation or median and interquartile range, according to the normality of the data. Normality, in turn, was verified by the Shapiro–Wilk test. All statistical analyses followed the intention-to-treat strategy. The primary outcome was the difference between the median VISA-G score at day thirty and baseline, tested by the Mann–Whitney test. To report the effect size of the main outcome, because the data (herein, the difference between post- and pre-intervention values) were nonparametric, the rank biserial correlation was calculated. The interaction between time and allocation was evaluated by a linear mixed model for repeated measures. In the model, the times (i.e., baseline, immediately after, day 30, and day 60) and the groups (active or control) were considered fixed-effect factors, and the individuals were considered random-effect factors. Therefore, baseline values were adjusted as they were treated as covariates. The parameters were estimated by full maximum likelihood, and the repeated-measures covariance was unstructured. All multiple comparisons were adjusted by the Bonferroni method. *p*-values ≤ 0.05 were considered statistically significant. The software used for the data analysis was SPSS, version 32.0.0.0 (IBM, Chicago, IL, USA).

## 3. Results

**Figure 1:**
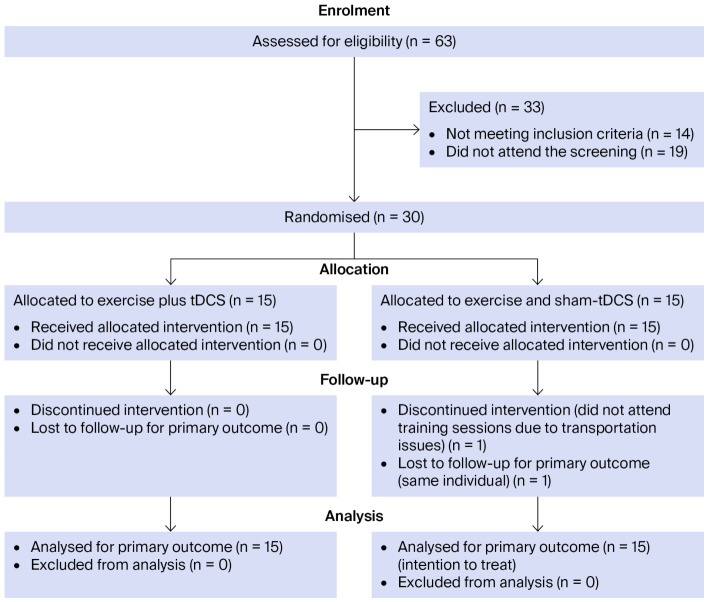
CONSORT flow diagram [13], depicting the inclusion details. tDCS: transcranial direct current stimulation.

The study was conducted between July 2023 and January 2025. As described in Section 2, people waiting for consultation at the outpatient clinics of a tertiary hospital were questioned about hip pain and subsequently evaluated according to the inclusion and exclusion criteria. These clinics serve approximately 30 patients per week, and all adult patients and family members in the waiting room were approached, consecutively. The inclusion flow is detailed in Figure 1.

A total of 30 participants were included in the study, according to the sample size calculation designed to achieve 80% power. The sample consisted of 8 men and 22 women, with a mean age of 53.9 (±12.1) years. There was only one dropout before the first exercise session due to personal reasons. The remaining participants completed 100% of the training sessions. The study concluded with 5.8% missing data overall, primarily due to one early dropout—a participant who underwent randomization but did not complete any outcome assessment—and occasional missing questionnaires, usually resulting from the inability to assess participants within the predefined timeframe for a given outcome. Based on the characteristics of the missingness, the data were considered compatible with a missing completely at random mechanism, and no imputation methods were applied. The baseline characteristics of the groups are provided in Table 1.

**Figure 2:**
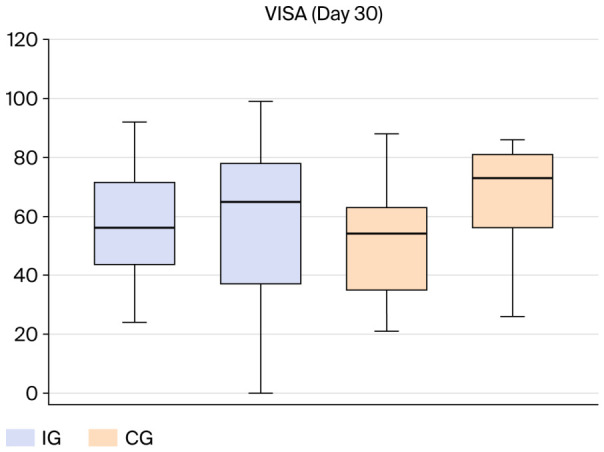
Primary outcome: VISA scores between groups at day thirty. The box plot presents the medians, interquartile ranges, and full ranges at baseline and at day thirty after the intervention. Participants who received active transcranial direct current electrical stimulation (tDCS) combined with resistance exercise showed numerically worse results than those in the control group (sham tDCS plus exercise), although this difference was not statistically significant (*p* = 0.460). VISA: Victorian Institute of Sport Assessment–Gluteal Tendinopathy, Brazilian version, ranging from 0 to 100, with higher scores indicating less pain and disability; IG: intervention group—tDCS combined with low-intensity resistance training; CG: control group—sham tDCS combined with low-intensity resistance training.

The sample consisted predominantly of middle-aged women, which is consistent with the known epidemiological profile of GTPS. The baseline values of the outcome measures indicate a moderate level of pain and functional limitation at the start of the study, consistent with the expected clinical presentation of this condition. Medical treatment remained unchanged in all but three patients (two in the intervention group and one in the control group), who used short courses of nonsteroidal anti-inflammatory drugs. The primary outcome, VISA, improved in both groups at day thirty, with the CG displaying slightly better results, although the difference was not statistically significant (ES −0.16, 95% CI −0.54 to 0.27, *p* = 0.460; Figure 2). Longitudinal repeated-measures analysis of VISA-G also showed no differences between groups at all time points (mean difference 1.54, 95% CI −12.02 to 15.12, *p* = 0.817). In addition, only the domain “Role Emotional” of SF-36 was significantly different between groups, favoring the intervention (mean difference 31.78, 95% CI 13.90 to 48.71, *p* = 0.013). In all other domains of all other outcomes, the tDCS plus exercise group performed no differently from the exercise alone group (Supplementary Table S1).

**Figure 3:**
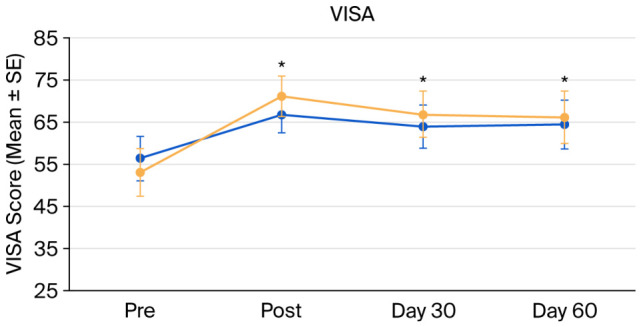
VISA performance over time among all participants (blue: intervention group; orange: control group). The figure illustrates the mean VISA scores analyzed using a linear mixed model with time and group as covariates. An improvement from baseline was observed in both groups and was sustained for up to sixty days. However, no difference was found between groups. SE: standard error; VISA: Victorian Institute of Sport Assessment–Gluteal Tendinopathy, Brazilian version, ranging from 0 to 100, with higher scores indicating less pain and disability. * Significant improvement relative to baseline (*p* < 0.05).

The effect of time was evaluated in the longitudinal analysis across multiple time points. It showed a consistent pattern of improvement in the scores of VISA, MPQ, PQAS, multiple domains of HAGOS, and some domains of SF-36 in the periods post-intervention and/or at day sixty (Table 2). Considering the main outcome, VISA, an improvement of 26% was obtained in the post-intervention period (*p* = 0.002), with a 19% improvement relative to baseline retained at day sixty (*p* = 0.021). The improvement, however, did not overcome the threshold of the minimal clinically important difference (i.e., 29%) [26]. Figure 3 illustrates these findings.

Considering other secondary outcomes, only the domains “General Health Perception”, “Role Limitations Due to Emotional Problems”, and “Social Functioning” of SF-36 demonstrated no significant effect of time. Both groups demonstrated improvements in at least one time point in all other metrics. The effect of time at the end of the study across all outcomes is summarized in Table 2, while the effect at every time point is provided in Supplementary Table S1.

Regarding safety and adherence, as mentioned earlier, one participant withdrew from the trial immediately after randomization. This individual reported that the decision was motivated by personal reasons—family caregiving—and was unrelated to the trial. Therefore, the adherence rate was 97%. We observed ten adverse events, all classified as grade 1 according to the CTCAE [22]: somnolence (n = 3, two in IG), headache (n = 2, both in IG), delayed-onset muscle soreness (DOMS; n = 4, two in IG), and depressive symptoms (n = 1, IG). Somnolence and headache are common side effects of tDCS and were attributed to neurostimulation. They were brief and of low intensity, allowing participants to continue the trial. In contrast, DOMS was related to the exercise protocol, whereas the depressive episode occurred in a participant with a previous diagnosis of depression; thus, causality could not be established.

## 4. Discussion

In this study, no positive effects were identified regarding the application of anodal motor cortex tDCS for GTPS. To the best of our knowledge, this is the first study to address this intervention. On the other hand, a positive and sustained effect was found for both groups over time in most of the outcomes analyzed, in spite of the short duration of the intervention. Although tDCS has been widely investigated and systematic reviews indicate its potential benefits for various pain conditions [8], the inability of this intervention to promote improvement in GTPS may be related to the complex and multifactorial nature of this condition, which is influenced by several factors, such as obesity, history of previous knee pathologies and low back pain [3], kinetic alterations (e.g., excessive hip adduction), muscle weakness, and excessive or inadequate use of the gluteal muscles [25]. In tDCS, the potential benefits are thought to be mediated by central mechanisms, as in fibromyalgia [9], and by peripheral mechanisms, as in knee osteoarthritis and myofascial syndrome [10]. However, the specific effect of tDCS combined with resisted physical exercise for musculoskeletal pain is not yet well established [28], which may explain our null findings for this particular condition.

Another factor that may have contributed to the lack of positive effects was the short stimulation duration. In the present study, we employed a very short protocol aiming to optimize adherence. Our choice of four days was based on previous studies using brief intervention periods [12,25,26] and our own pilot study [14]. However, while this strategy minimized absenteeism, it may also have reduced efficacy. Greater effects might have been observed if the stimulation had been conducted for a longer period, whether more days or weeks. This remains a potential subject for future investigation.

The choice of current intensity and application time was also based on previous studies on tDCS, alone or in combination with physical exercise. For example, Zhu et al. evaluated six studies (totaling 192 participants) of tDCS in patients with fibromyalgia, in which the applied current was 2 mA, with a number of sessions ranging from one to ten, performed daily or weekly, lasting 20 min each [9]. Similarly, Souza et al. investigated the combination of tDCS and physical exercise in three clinical trials totaling 110 participants [28]. In that study, the current intensity varied between one and 2 mA, with sessions performed daily or every other day, totaling between five and sixteen sessions, also lasting 20 min each. Reported side effects were mild and did not differ significantly from the placebo group [28]. Therefore, while there may be room for different currents and application times to be tested in GTPS, the current evidence remains inconclusive.

Another aspect to consider is related to the site of application. The choice of anodal stimulation over the primary motor cortex aligns with most studies addressing pain [29,30,31]. However, the dorsolateral prefrontal cortex has increasingly gained attention [29], with some studies suggesting even greater efficacy in comparison with M1 for specific pain conditions, such as migraine [32], and for adding a sensory and affective modulation component [33].

A further limitation of the present study was the absence of an objective anatomical parameter, such as ultrasound (US) or magnetic resonance imaging (MRI). The accuracy of MRI for detecting tendinopathy and bursitis is very high (approximately 91%) [34], and although bursitis is not necessarily common in GTPS [4], it is sensitive to inflammatory changes and can be detected in fluid-sensitive sequences (such as T2). US could also assess textural and architectural changes involving the gluteal tendons [35], allowing for serial evaluations. Interrater variability may pose a challenge, but combining imaging with clinical data could provide additional insight into the factors related to improvement.

Although no substantial benefits were observed with the application of tDCS, improvements were identified when assessing the effect of exercise on GTPS over time. While this could be merely due to the placebo effect—e.g., participants improved because they imagined they were stimulated or because they felt cared for—the possible contribution of the exercise protocol—which was ultimately applied equally to all volunteers—cannot be ignored. According to Booth et al. and Meade et al., physical exercise has been widely prescribed for the management of musculoskeletal pain through a mechanism known as exercise-induced hypoalgesia [36,37]. This mechanism is believed to be regulated by the release of endogenous opioids in response to increased motor cortex activity [38]. Furthermore, exercise-induced hypoalgesia can improve musculoskeletal performance and function [36]. There is evidence to suggest that aerobic and resistance exercises are effective in reducing pain in patients with fibromyalgia [39,40,41].

In the present intervention, a conservative treatment protocol adapted from the GLoBE protocol [23] was implemented to produce a low-intensity workout, allowing inclusion of individuals with severe impairment. Therefore, the findings of this study could contribute to the recommendations of French and colleagues, who highlighted that the most effective approach to GTPS treatment involves exercise prescription [42]. These results reinforce the importance of well-structured rehabilitation strategies in the management of GTPS. As most exercises were simple and required minimal equipment, future research may assess whether home-based strategies [43] can produce similar effects.

### Limitations of the Study

This study has several limitations (some of them partially explored previously). First, the short intervention time may not have been sufficient to promote the analgesic and neuroplastic changes we speculated, as stimulation was administered for only four days. In addition, the absence of a true placebo arm, that is, a group that did not exercise, hampered the conclusions about the true effectiveness of the exercise protocol. Trials with more neurostimulation sessions and the inclusion of a no-exercise control group could help to clarify these issues. Another limitation is the absence of an objective radiological parameter for diagnosis and improvement. The implementation of US or MRI to diagnose and follow GTPS could guarantee more homogeneity in the inclusion process, and the analysis of radiological parameters as outcomes could help to better understand the mechanisms underlying the patient-reported outcomes. Finally, the study included multiple outcomes, multiple domains, and multiple follow-up time points, so the interpretation of the results may be misleading. Nevertheless, the primary endpoint was based on a single-domain outcome measured at a specific time point, with nonsignificant results; therefore, type I error inflation should not be a major concern. Secondary outcomes are more prone to multiplicity and, despite the Bonferroni adjustment for type I error, should be regarded as exploratory only.

## 5. Conclusions

We can conclude that the application of anodal motor cortex tDCS combined with resistance exercise for GTPS was no different from the application of exercise alone. However, both groups improved significantly, suggesting a possible role of the exercise protocol, which was short in duration and low in intensity. While we suggest that tDCS should not be used for GTPS outside research settings, studies assessing whether stimulation protocols with a larger number of sessions or different stimulation parameters can produce similar effects are warranted.

## Figures and Tables

**Table 1:** Baseline characteristics of the groups.

	Domain	tDCS + RE (n = 15)	RE Only (n = 15)	*p*-Value
Age, years	N/A	55 ± 12.7	53 ± 11.8	0.606
Female sex, n	N/A	11/15	11/15	1
VISA	N/A	56 (44–72)	54 (35–63)	0.644
MPQ	PRI	31.3 ± 11	32.1 ± 7.3	0.827
HAGOS	Symptoms	44.6 ± 13.9	48.8 ± 12.9	0.425
	Stiffness	49.1 ± 24.2	56.7 ± 23.7	0.417
	Pain	43.7 ± 19.2	49.0 ± 20.0	0.491
	Daily living	41.7 ± 18.4	48.0 ± 20.8	0.414
	S&R	44.4 ± 30.4	34.6 ± 16.1	0.303
	PPA	34.8 ± 27.3	38.4 ± 20.0	0.699
	Quality of life	36.4 ± 21.6	33.8 ± 18.6	0.743
PQAS	Paroxysmal	5.2 ± 2.5	4.9 ± 2.4	0.756
	Superficial	3.7 ± 3.0	4.7 ± 2.3	0.347
	Deep	4.8 ± 2.5	4.7 ± 2.3	0.912
SF-36	PF	55 (27.5–63.7)	30 (15–55)	0.305
	RP	37.5 (0–93.7)	0 (0–25)	0.072
	Pain	41 (31–48.5)	41 (22–51)	0.62
	GH	64.2 ± 21.0	52.0 ± 25.9	0.19
	Vitality	31.7 ± 18.0	30.0 ± 15.6	0.787
	SF	60.7 ± 31.3	56.7 ± 30.0	0.739
	REm	50 (8.3–100)	0 (0–33.3)	0.078
	Mental health	62.0 ± 23.2	55.0 ± 18.5	0.402

Data are expressed as mean ± standard deviation or median (interquartile range). GH: general health perception; HAGOS: Copenhagen Hip and Groin Outcome Score, Brazilian version, ranging from 0 to 100 across six domains, with higher scores indicating better outcomes; MPQ: McGill Pain Questionnaire, Brazilian version, ranging from 0 to 78, with higher scores indicating greater pain intensity (worse condition); PF: physical functioning; PPA: participation in physical activities; PQAS: Pain Quality Assessment Scale, Brazilian version, ranging from 0 to 10, with higher scores indicating more intense pain; PRI: pain rating index; RE: low-intensity resistance exercise; REm: role limitations due to emotional problems; RP: role limitations due to physical health; S&R: sports and recreation; SF: social functioning; SF-36: Medical Outcomes Short-Form Health Survey, Brazilian version, which assesses quality of life across multiple domains, with higher values associated with better quality of life; tDCS: transcranial direct current electrical stimulation; VISA: Victorian Institute of Sport Assessment—Gluteal Tendinopathy, Brazilian version, which ranges from 0 to 100, with 100 representing the best possible result.

**Table 2:** Longitudinal analysis of the effect of time at the end of follow-up relative to baseline.

Outcome	Domain	Estimate (CI)	*p*-Value
VISA	N/A	10.61 (1.17 to 20.04)	**0.021**
HAGOS	Symptoms	12.33 (3.49 to 21.17)	**0.003**
	Stiffness	11.50 (1.18 to 21.82)	**0.022**
	Pain	15.67 (4.29 to 27.04)	**0.003**
	Daily living	15.23 (5.34 to 25.11)	**<0.001**
	S&R	14.65 (−0.31 to 29.62)	0.058
	PPA	15.24 (1.38 to 29.11)	**0.025**
	Quality of life	16.34 (5.14 to 27.55)	**0.002**
PQAS	Paroxysmal	−2.02 (−2.99 to −1.06)	**<0.001**
	Superficial	−1.54 (−2.50 to −0.57)	**<0.001**
	Deep	−1.95 (−3.01 to −0.89)	**<0.001**
MPQ	Total (PRI)	−6.854 (−1.35 to −12.34)	**0.008**
SF-36	PF	16.23 (8.45 to 24.01)	**<0.001**
	RP	10.81 (−10.36 to 31.99)	0.951
	Pain	16.05 (6.82 to 25.27)	**<0.001**
	GH	2.31 (−4.46 to 9.09)	1
	Vitality	8.53 (1.74 to 15.33)	**0.008**
	SF	11.61 (−0.53 to 23.75)	0.067
	RE	14.17 (−10.50 to 38.86)	0.684
	Mental health	7.98 (1.73 to 14.24)	**0.007**

Data are expressed as mean differences (95% CI) and *p*-values. Significant *p*-values are bolded. CI: confidence interval; GH: general health perception; HAGOS: Copenhagen Hip and Groin Outcome Score, Brazilian version; MPQ: McGill Pain Questionnaire, Brazilian version; PF: physical functioning; PPA: participation in physical activities; PQAS: Pain Quality Assessment Scale, Brazilian version; PRI: Pain rating index; RE: role limitations due to emotional problems; RP: role limitations due to physical health; S&R: sports and recreation; SF: social functioning; SF-36: Medical Outcomes Short-Form Health Survey-36, Brazilian version; VISA: Victorian Institute of Sport Assessment—Gluteal Tendinopathy, Brazilian version.

## Data Availability

The data presented in the study are openly available in Repositório de Dados de Pesquisa da Unicamp (REDU) at https://doi.org/10.25824/redu/RTEK2K (accessed on 9 June 2026).

## Supplementary Materials

The following supporting information can be downloaded at: https://www.mdpi.com/article/10.3390/medsci14020312/s1, Supplementary File S1: Pre-screening questionnaire for the recruitment of the trial; Supplementary File S2: Exercises; Supplementary Table S1: Synthesis of the results of the linear mixed model assessing the effect of group allocation and time.

## Abbreviations

The following abbreviations are used in this manuscript:

CG	Control group
CTCAE	Common Terminology Criteria for Adverse Events
DOMS	Delayed-onset muscle soreness
GTPS	Greater trochanteric pain syndrome
HAGOS	Copenhagen Hip and Groin Outcome Score, Brazilian version
IG	Intervention group
MPQ	McGill Pain Questionnaire, Brazilian version
MRI	Magnetic resonance imaging
PQAS	Pain Quality Assessment Scale, Brazilian version
RE	Resistance exercise
REBEC	Registro Brasileiro de Ensaios Clínicos
SF-36	Short-Form Health Survey, Brazilian version
tDCS	Transcranial direct current electrical stimulation
US	Ultrasound
VISA	Victorian Institute of Sports Assessment–Gluteal Tendinopathy, Brazilian version

## References

[1] Grimaldi A., Mellor R., Hodges P., Bennell K., Wajswelner H., Vicenzino B. (2015). Gluteal Tendinopathy: A Review of Mechanisms, Assessment and Management. Sports Med..

[2] Segal N.A., Felson D.T., Torner J.C., Zhu Y., Curtis J.R., Niu J., Nevitt M.C. (2007). Greater trochanteric pain syndrome: Epidemiology and associated factors. Arch. Phys. Med. Rehabil..

[3] Fearon A.M., Scarvell J.M., Neeman T., Cook J.L., Cormick W., Smith P.N. (2013). Greater trochanteric pain syndrome: Defining the clinical syndrome. Br. J. Sports Med..

[4] Bird P.A., Oakley S.P., Shnier R., Kirkham B.W. (2001). Prospective evaluation of magnetic resonance imaging and physical examination findings in patients with greater trochanteric pain syndrome. Arthritis Rheumatol..

[5] Kong A., Van der Vliet A., Zadow S. (2007). MRI and US of gluteal tendinopathy in greater trochanteric pain syndrome. Eur. Radiol..

[6] Mellor R., Bennell K., Grimaldi A., Nicolson P., Kasza J., Hodges P., Wajswelner H., Vicenzino B. (2018). Education plus exercise versus corticosteroid injection use versus a wait and see approach on global outcome and pain from gluteal tendinopathy: Prospective, single blinded, randomised clinical trial. Br. J. Sports Med..

[7] Thair H., Holloway A.L., Newport R., Smith A.D. (2017). Transcranial Direct Current Stimulation (tDCS): A Beginner’s Guide for Design and Implementation. Front. Neurosci..

[8] Pinto C.B., Teixeira Costa B., Duarte D., Fregni F. (2018). Transcranial Direct Current Stimulation as a Therapeutic Tool for Chronic Pain. J. ECT.

[9] Zhu C.E., Yu B., Zhang W., Chen W.H., Qi Q., Miao Y. (2017). Effiectiveness and safety of transcranial direct current stimulation in fibromyalgia: A systematic review and meta-analysis. J. Rehabil. Med..

[10] Cardenas-Rojas A., Pacheco-Barrios K., Giannoni-Luza S., Rivera-Torrejon O., Fregni F. (2020). Noninvasive brain stimulation combined with exercise in chronic pain: A systematic review and meta-analysis. Expert Rev. Neurother..

[11] Teixeira P.E.P., Alawdah L., Alhassan H.A.A., Guidetti M., Priori A., Papatheodorou S., Fregni F. (2020). The Analgesic Effect of Transcranial Direct Current Stimulation (tDCS) combined with Physical Therapy on Common Musculoskeletal Conditions: A Systematic Review and Meta-Analysis. Princ. Pract. Clin. Res..

[12] Saucedo Marquez C.M., Zhang X., Swinnen S.P., Meesen R., Wenderoth N. (2013). Task-specific effect of transcranial direct current stimulation on motor learning. Front. Hum. Neurosci..

[13] Hopewell S., Chan A.W., Collins G.S., Hróbjartsson A., Moher D., Schulz K.F., Tunn R., Aggarwal R., Berkwits M., Berlin J.A. (2025). CONSORT 2025 Statement: Updated Guideline for Reporting Randomized Trials. JAMA.

[14] Martins E.F., Carneiro J.A., Ribeiro C.T., Carreño P.G., Okumura N.L., de Souza J.M. (2025). Effect of transcranial direct current electric stimulation associated with physical exercise in patients with greater trochanteric pain syndrome. Med. Case Rep. Study Protoc..

[15] Grimaldi A., Mellor R., Nicolson P., Hodges P., Bennell K., Vicenzino B. (2017). Utility of clinical tests to diagnose MRI-confirmed gluteal tendinopathy in patients presenting with lateral hip pain. Br. J. Sports Med..

[16] Tan L.A., Benkli B., Tuchman A., Li X.J., Desai N.N., Bottiglieri T.S., Pavel J., Lenke L.G., Lehman R.A. (2018). High prevalence of greater trochanteric pain syndrome among patients presenting to spine clinic for evaluation of degenerative lumbar pathologies. J. Clin. Neurosci..

[17] Paiva E.B., Azevedo D.C., Pereira A.L., Garcia A.N., Percope de Andrade M.A. (2021). Translation, cross-cultural adaptation and validation of the Brazilian Portuguese version of the Victorian Institute of Sports Assessment for Gluteal Tendinopathy patient reported-outcome measure (VISA-G.BR). Musculoskelet. Sci. Pract..

[18] Mendonça L.M., Camelo P.R.P., Trevisan G.C.C., Bryk F.F., Thorborg K., Oliveira R.R. (2021). The Brazilian Hip and Groin Outcome Score (HAGOS-Br): Cross-cultural adaptation and measurement properties. Braz. J. Phys. Ther..

[19] Carvalho A.B., Garcia J.B., Silva T.K., Ribeiro J.V. (2016). Translation and transcultural adaptation of Pain Quality Assessment Scale (PQAS) to Brazilian version. Braz. J. Anesthesiol..

[20] Varoli F.K., Pedrazzi V. (2006). Adapted version of the McGill Pain Questionnaire to Brazilian Portuguese. Braz. Dent. J..

[21] Ciconelli R., Ferraz M., Santos W., Meinao I., Quaresma M. (1999). Brazilian-Portuguese version of the SF-36. A reliable and valid quality of life outcome measure. Rev. Bras. Reumatol..

[22] NIH Common Terminology Criteria for Adverse Events (CTCAE) Version 5.0. https://ctep.cancer.gov/protocolDevelopment/electronic_applications/docs/CTCAE_v5_Quick_Reference_5x7.pdf.

[23] Ganderton C., Semciw A., Cook J., Moreira E., Pizzari T. (2018). Gluteal Loading Versus Sham Exercises to Improve Pain and Dysfunction in Postmenopausal Women with Greater Trochanteric Pain Syndrome: A Randomized Controlled Trial. J. Women’s Health.

[24] Klem G.H., Lüders H.O., Jasper H.H., Elger C. (1999). The ten-twenty electrode system of the International Federation. The International Federation of Clinical Neurophysiology. Electroencephalogr. Clin. Neurophysiol. Suppl..

[25] Sakrajai P., Janyacharoen T., Jensen M.P., Sawanyawisuth K., Auvichayapat N., Tunkamnerdthai O., Keeratitanont K., Auvichayapat P. (2014). Pain reduction in myofascial pain syndrome by anodal transcranial direct current stimulation combined with standard treatment: A randomized controlled study. Clin. J. Pain.

[26] Misse R.G., Dos Santos A.M., De Souza J.M., Shinjo S.K. (2020). Transcranial direct current stimulation improves myofascial pain syndrome and chronic fatigue. Reumatismo.

[27] Ebert J.R., Fearon A.M., Smith A., Janes G.C. (2019). Responsiveness of the Victorian Institute for Sport Assessment for Gluteal Tendinopathy (VISA-G), modified Harris hip and Oxford hip scores in patients undergoing hip abductor tendon repair. Musculoskelet. Sci. Pract..

[28] Souza R.V.S., Maciel D.G., Cerqueira M.S. (2021). Effects of transcranial direct current stimulation associated or combined with exercise on musculoskeletal pain: Systematic review. BrJP.

[29] Pacheco-Barrios K., Cardenas-Rojas A., Thibaut A., Costa B., Ferreira I., Caumo W., Fregni F. (2020). Methods and strategies of tDCS for the treatment of pain: Current status and future directions. Expert Rev. Med. Devices.

[30] Jiang X., Wang Y., Wan R., Feng B., Zhang Z., Lin Y., Wang Y. (2022). The effect of high-definition transcranial direct current stimulation on pain processing in a healthy population: A single-blinded crossover controlled study. Neurosci. Lett..

[31] Lefaucheur J.-P., Antal A., Ayache S.S., Benninger D.H., Brunelin J., Cogiamanian F., Cotelli M., De Ridder D., Ferrucci R., Langguth B. (2017). Evidence-based guidelines on the therapeutic use of transcranial direct current stimulation (tDCS). Clin. Neurophysiol..

[32] Andrade S.M., de Brito Aranha R.E.L., de Oliveira E.A., de Mendonça C.T.P.L., Martins W.K.N., Alves N.T., Fernández-Calvo B. (2017). Transcranial direct current stimulation over the primary motor vs. prefrontal cortex in refractory chronic migraine: A pilot randomized controlled trial. J. Neurol. Sci..

[33] Sankarasubramanian V., Cunningham D.A., Potter-Baker K.A., Beall E.B., Roelle S.M., Varnerin N.M., Machado A.G., Jones S.E., Lowe M.J., Plow E.B. (2017). Transcranial Direct Current Stimulation Targeting Primary Motor Versus Dorsolateral Prefrontal Cortices: Proof-of-Concept Study Investigating Functional Connectivity of Thalamocortical Networks Specific to Sensory-Affective Information Processing. Brain Connect..

[34] Cvitanic O., Henzie G., Skezas N., Lyons J., Minter J. (2004). MRI diagnosis of tears of the hip abductor tendons (gluteus medius and gluteus minimus). AJR Am. J. Roentgenol..

[35] Connell D.A., Bass C., Sykes C.A., Young D., Edwards E. (2003). Sonographic evaluation of gluteus medius and minimus tendinopathy. Eur. Radiol..

[36] Booth J., Moseley G.L., Schiltenwolf M., Cashin A., Davies M., Hübscher M. (2017). Exercise for chronic musculoskeletal pain: A biopsychosocial approach. Musculoskelet. Care.

[37] Meade L.B., Bearne L.M., Sweeney L.H., Alageel S.H., Godfrey E.L. (2019). Behaviour change techniques associated with adherence to prescribed exercise in patients with persistent musculoskeletal pain: Systematic review. Br. J. Health Psychol..

[38] Mendonca M.E., Simis M., Grecco L.C., Battistella L.R., Baptista A.F., Fregni F. (2016). Transcranial Direct Current Stimulation Combined with Aerobic Exercise to Optimize Analgesic Responses in Fibromyalgia: A Randomized Placebo-Controlled Clinical Trial. Front. Hum. Neurosci..

[39] Sosa-Reina M.D., Nunez-Nagy S., Gallego-Izquierdo T., Pecos-Martín D., Monserrat J., Álvarez-Mon M. (2017). Effectiveness of Therapeutic Exercise in Fibromyalgia Syndrome: A Systematic Review and Meta-Analysis of Randomized Clinical Trials. Biomed. Res. Int..

[40] Nicolson P.J.A., Bennell K.L., Dobson F.L., Van Ginckel A., Holden M.A., Hinman R.S. (2017). Interventions to increase adherence to therapeutic exercise in older adults with low back pain and/or hip/knee osteoarthritis: A systematic review and meta-analysis. Br. J. Sports Med..

[41] Wilson F., Walshe M., O’Dwyer T., Bennett K., Mockler D., Bleakley C. (2018). Exercise, orthoses and splinting for treating Achilles tendinopathy: A systematic review with meta-analysis. Br. J. Sports Med..

[42] French H.P., Jong C.C., McCallan M. (2019). Do features of central sensitisation exist in Greater Trochanteric Pain Syndrome (GTPS)? A case control study. Musculoskelet. Sci. Pract..

[43] Silva V.S.X., Rivera R.J.B., Martins E.F., Uchida M.C., de Souza J.M. (2025). The Role of Home-Based Exercise in Managing Common Musculoskeletal Disorders: A Narrative Review. J. Funct. Morphol. Kinesiol..

