# Supplementary material for: Transcranial Direct Current Electric Stimulation Combined with Physical Exercise in Patients with Greater Trochanteric Pain Syndrome: Randomized Clinical Trial
Source: Med Sci (Basel). 2026 Jun 12;14(2):312. doi: 10.3390/medsci14020312 (PMC13304131; doi:10.3390/medsci14020312)

*Supplementary files*

*Supplementary File S1*

This is a translated version (the original is in Brazilian Portuguese) of the form provided to the researchers during participant recruitment for the study.

# Pre-screening Questionnaire

**1. Does the volunteer have hip pain?**

☐ Yes
☐ No

**2. Ask whether the pain is truly located in the hip region (i.e., whether it begins in the groin, greater trochanter/tensor fasciae latae region, or lateral gluteal region). Sequentially point to these three regions.**

☐ The pain is truly located in the hip region
☐ The pain is not located in the hip region

**3. Has the pain been present for more than three months?**

☐ Yes
☐ No

**4. Ask whether the pain occurs in one or more of the following situations: while going up or down slopes or stairs; when lying on the affected side; when pressure is applied (to touch); when sitting down or standing up from a chair or toilet.**

☐ Yes, it occurs in at least one of these situations
☐ No, it does not occur in at least one of these situations

**Please invite the volunteer for the physical examination only if all answers are affirmative.**

*Supplementary Table S1*

*Synthesis of the results of the linear mixed model assessing the effect of group allocation and time.*

| Metric | **VISA** | **MPQ** | **PQAS** | | |
|---|---|---|---|---|---|
| Domain | N/A | PRI | Paroxysmal | Superficial | Deep |
| Mean difference | 1.549 | 3.781 | 0.546 | -0.069 | 0.526 |
| Lower CI | -12.022 | -3.703 | -1.201 | -1.74 | -1.121 |
| Higher CI | 15.120 | 11.266 | 2.294 | 1.602 | 2.172 |
| p value | 0.817 | 0.31 | 0.528 | 0.933 | 0.519 |
| Significance (EoT) | **0.003** | **<0.001** | **<0.001** | **<0.001** | **<0.001** |
| pre-post | **0.002** | **<0.001** | **<0.001** | **0.004** | **<0.001** |
| pre-30 | **0.041** | 1 | **<0.001** | **0.007** | **<0.001** |
| pre-60 | **0.021** | **0.008** | **<0.001** | **<0.001** | **<0.001** |
| post-30 | 0.777 | **<0,001** | 1 | 1 | 1 |
| post-60 | 0.922 | **0.006** | 1 | 1 | 1 |
| 30-60 | 1 | **0.034** | 1 | 0.217 | 1 |

| Metric | **HAGOS** | | | | | | |
|---|---|---|---|---|---|---|---|
| Domain | Symptoms | Stiffness | Pain | Daily living | S&R | PPA | Quality of life |
| Mean difference | 2.996 | 3.172 | 1.931 | 2.386 | -3.595 | 1.22 | -5.642 |
| Lower CI | -10.249 | -11.037 | -11.73 | -13.266 | -20.554 | -13.603 | -21.175 |
| Higher CI | 16.24 | 17.381 | 15.593 | 18.039 | 13.365 | 16.042 | 9.891 |
| p value | 0.646 | 0.651 | 0.774 | 0.757 | 0.667 | 0.867 | 0.463 |
| Significance (EoT) | **<0.001** | **0.026** | **<0.001** | **<0.001** | **0.003** | **0.015** | **0.001** |
| pre-post | **<0.001** | 0.052 | **0.003** | **<0.001** | **0.003** | 0.061 | **0.008** |
| pre-30 | **0.025** | 0.077 | 0.056 | **0.003** | 0.068 | 0.106 | **0.027** |
| pre-60 | **0.003** | **0.022** | **0.003** | **<0.001** | 0.058 | **0.025** | **0.002** |
| post-30 | 0.224 | 1 | 0.111 | 0.994 | 0.175 | 1 | 1 |
| post-60 | 1 | 1 | 1 | 1 | 1 | 1 | 1 |
| 30-60 | 1 | 1 | 1 | 1 | 1 | 1 | 0.433 |

| Metric | **SF-36** | | | | | | | |
|---|---|---|---|---|---|---|---|---|
| Domain | PF | RP | Pain | GH | Vitality | SF | RE | Mental health |
| Mean difference | -10.482 | -25.064 | -10.719 | -8.831 | -7.396 | -8.465 | -31,784 | -8.304 |
| Lower CI | -27.482 | -49.916 | -24.364 | -23.232 | -18.83 | -25.94 | -56.369 | -23.386 |
| Higher CI | 6.518 | -0.213 | 2.926 | 5.569 | 4.037 | 9.010 | -7.199 | 6.778 |
| p value | 0.217 | 0.048 | 0.119 | 0.219 | 0.196 | 0.330 | **0.013** | 0.269 |
| Significance (EoT) | **<0.001** | **0.006** | **<0.001** | 0.812 | **0.012** | **0.02** | 0.456 | **0.011** |
| pre-post | **0.023** | 0.26 | **0.01** | 1 | 1 | 0.126 | 1 | **0.025** |
| pre-30 | **0.007** | **0.007** | **0.004** | 1 | 0.485 | 1 | 1 | 0.387 |
| pre-60 | **<0.001** | 0.951 | **<0.001** | 1 | **0.008** | 0.067 | 0.684 | **0.007** |
| post-30 | 1 | 0.697 | 1 | 1 | 1 | 0.196 | 1 | 1 |
| post-60 | 0.148 | 1 | 1 | 1 | 0.179 | 1 | 1 | 1 |

| | | | | | | | | |
|---|---|---|---|---|---|---|---|---|
| 30-60 | 0.753 | 0.609 | 0.692 | 1 | 0.119 | 0.379 | 1 | 1 |

*CI: confidence interval; EoT: effect of time in the linear mixed model; GH: general health perception; HAGOS: Copenhagen Hip and Groin Outcome Score, Brazilian version, ranging from 0 to 100 across six domains, with higher scores indicating better outcomes; MPQ: McGill Pain Questionnaire, Brazilian version, ranging from 0 to 78, with higher scores indicating greater pain intensity (worse condition); PF: physical functioning; PPA: participation in physical activities; PQAS: Pain Quality Assessment Scale, Brazilian version, ranging from 0 to 10, with higher scores indicating more intense pain; PRI: Pain rating index; RE: low-intensity resistance exercise; RE: role limitations due to emotional problems; RP: role limitations due to physical health; S&R: sports and recreation; SF: social functioning; SF-36: Medical Outcomes Short-Form Health Survey, Brazilian version, which assesses quality of life across multiple domains, with higher values being associated with a better quality of life; tDCS: transcranial direct current electrical stimulation; VISA: Victorian Institute of Sport Assessment – Gluteal Tendinopathy, Brazilian version, which ranges from 0 to 100, with 100 representing the best result.*

*Supplementary File S2*

*Exercises*

All participants in the trial followed the same training protocol, described below.

1) Squat (to 60 Degrees of Knee Flexion), three sets of 10 repetitions, with 30 seconds of rest in between:

The participant stands with the feet shoulder-width apart and the toes slightly turned outward. He/She lowers the hips back and down as if sitting in a chair, bending the knees to approximately sixty degrees, while keeping the chest upright and the knees aligned with the toes. The movement ends by pushing through the heels to return to standing.

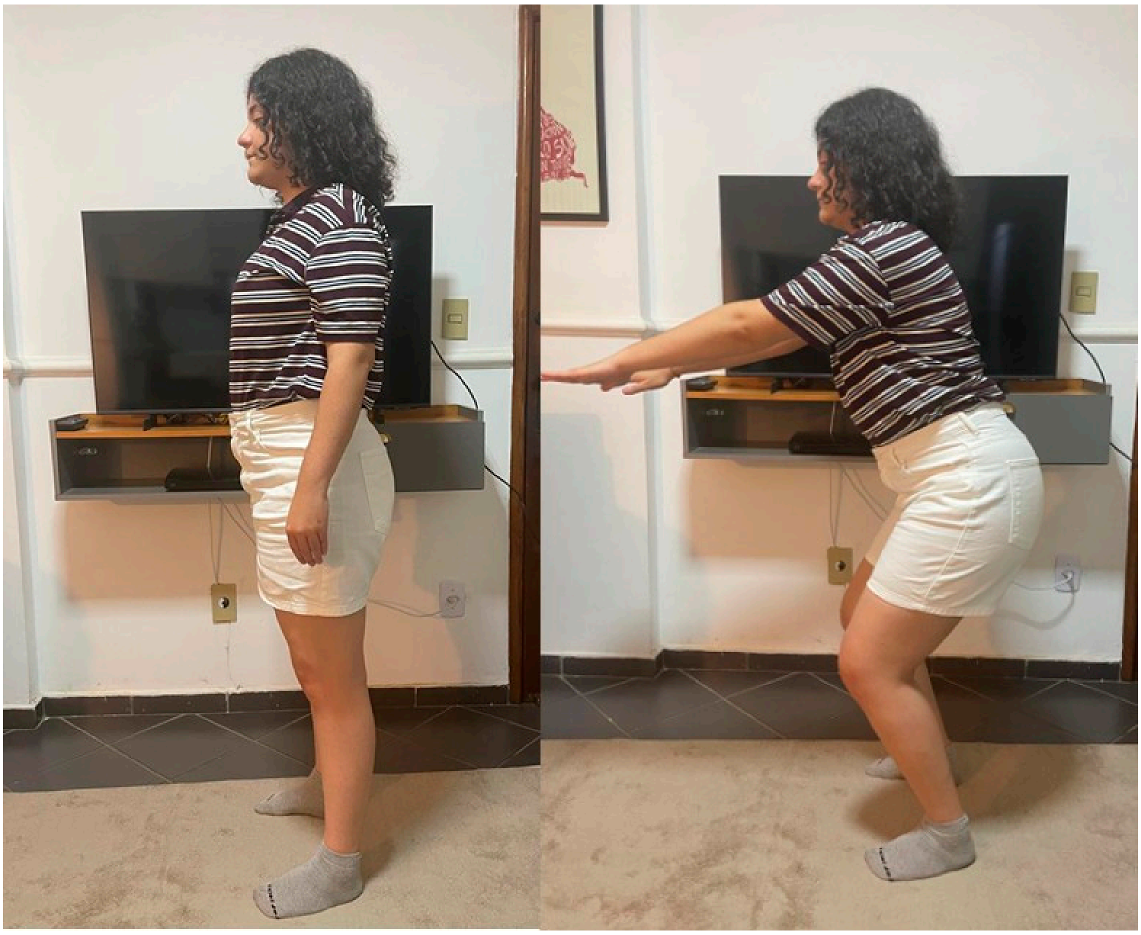

2) Single-leg support, five seconds on each leg, alternating, for 10 repetitions, done two times, with 30 seconds of rest in between:

The participant stands upright and lifts one foot off the ground, maintaining balance on the opposite leg. The position is held for five seconds, after which the participant returns both feet to the floor. The same procedure is then performed on the other leg. This alternation between legs continues until ten repetitions are completed (i.e., five on each leg).

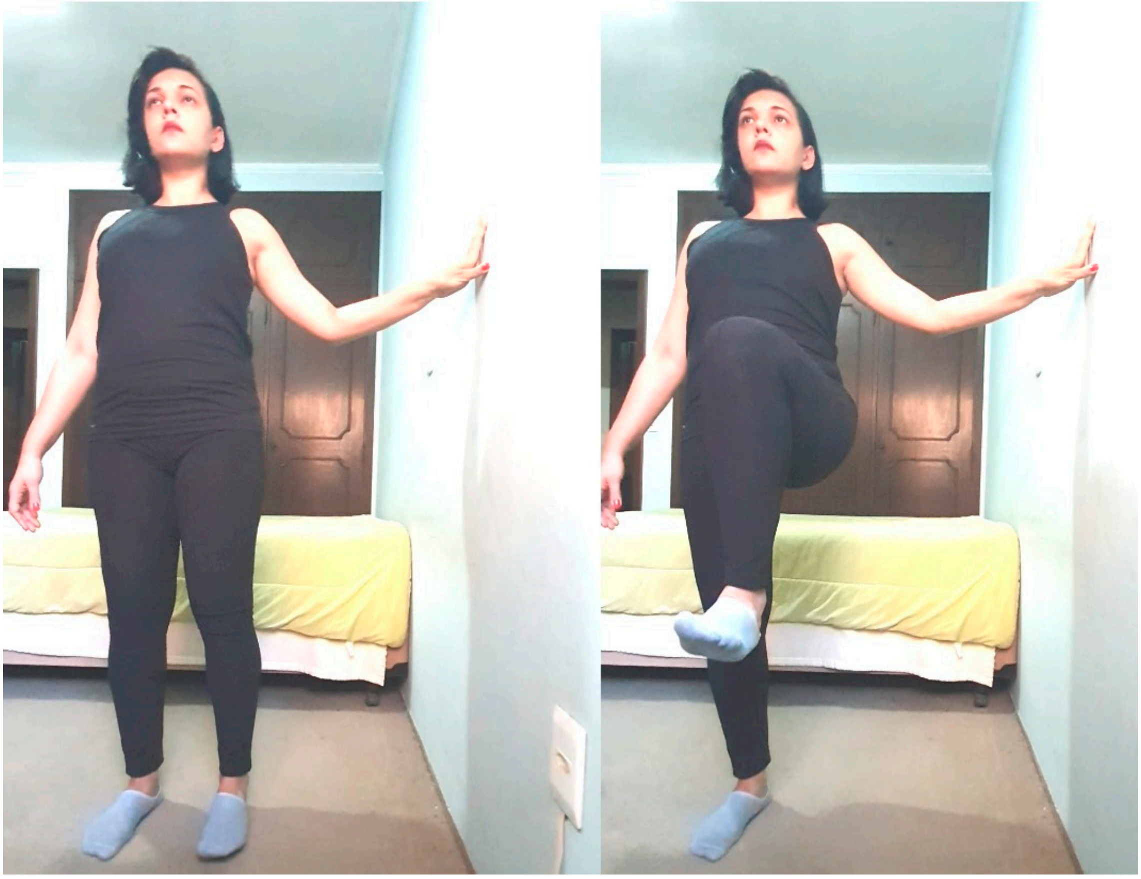

3) Step-Up, 10 repetitions on each limb, for three sets, with 30 seconds of rest between sets:

The participant places one foot onto a bench or box and pushes through the heel to stand fully on the elevated surface, then lowers back down in a controlled manner to the starting position.

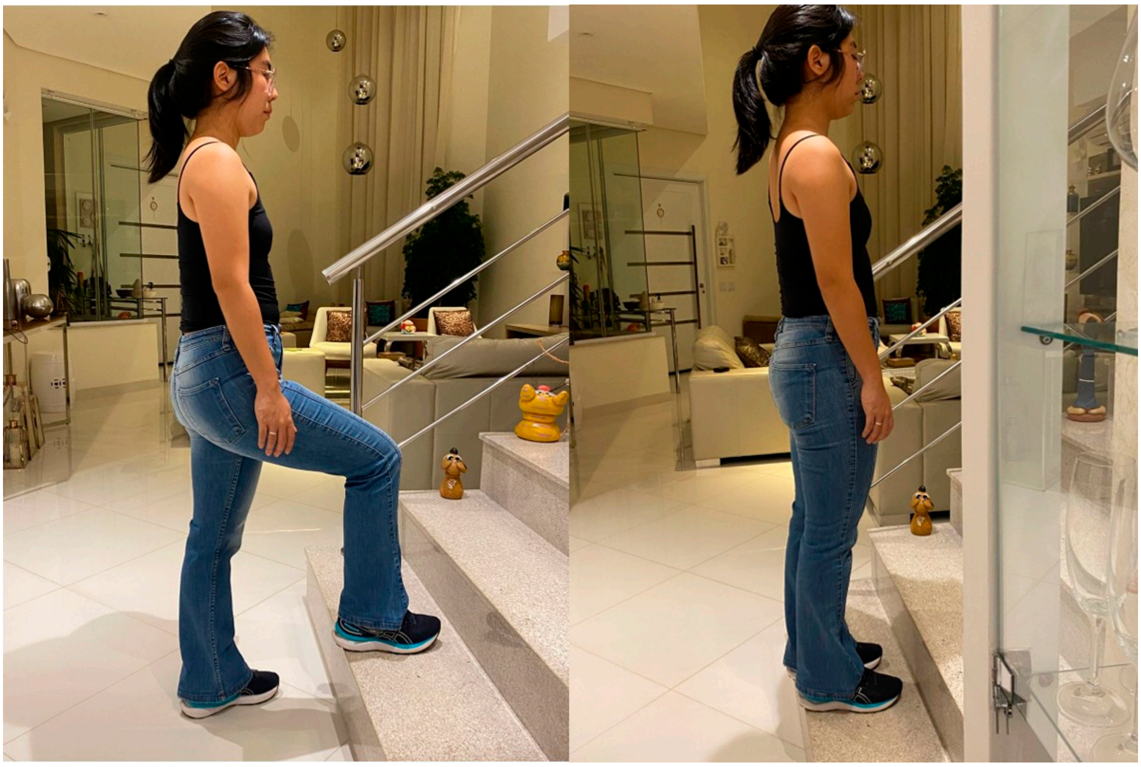

4) Hip raises, 10 repetitions, for three sets, with 30 seconds of rest between sets:

The participant lies on the back with the knees bent and the feet flat on the ground. He/She lifts the hips until forming a straight line from shoulders to knees, contracting the gluteal muscles at the top, and then lowers the hips slowly and with control to the initial position.

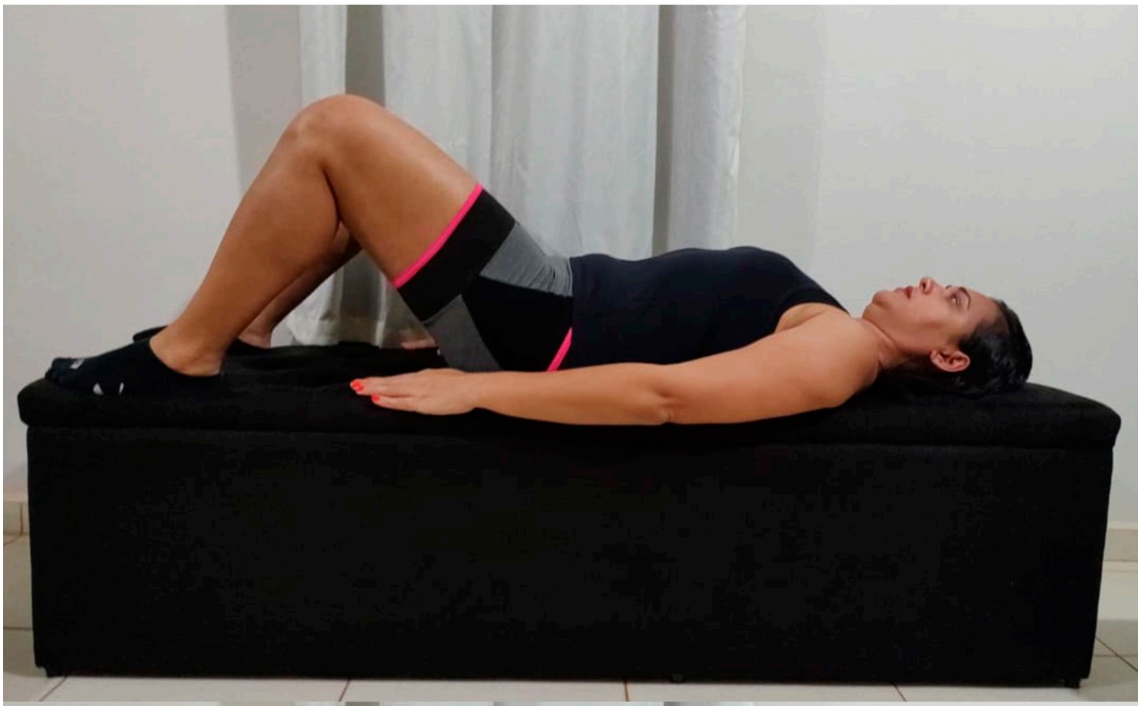

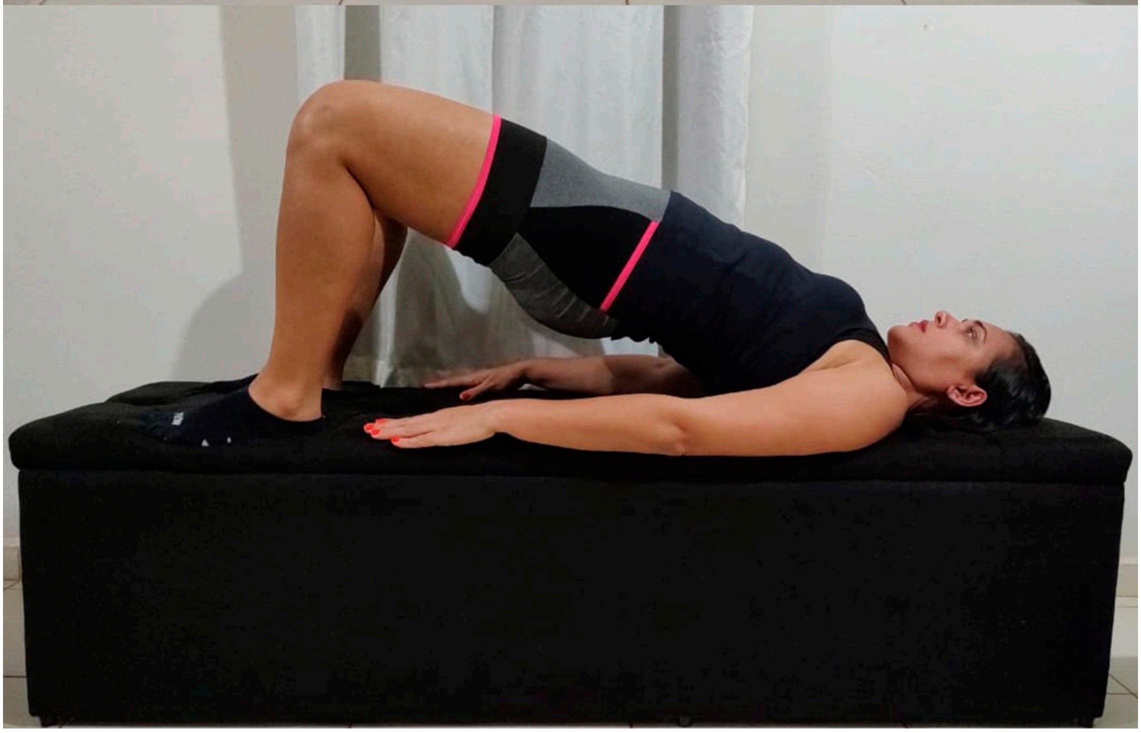

5) Seated abduction, resisted by an elastic band, 12 repetitions, for three sets, with a rest of 30 seconds between sets:

The participant sits with the feet flat on the floor and the thighs fully adducted. A resistance band is positioned around both thighs, just above the knees. The participant then abducts the thighs, moving the legs outward until the band is fully stretched. After a brief hold at the end range, the participant returns slowly and with control to the starting position.

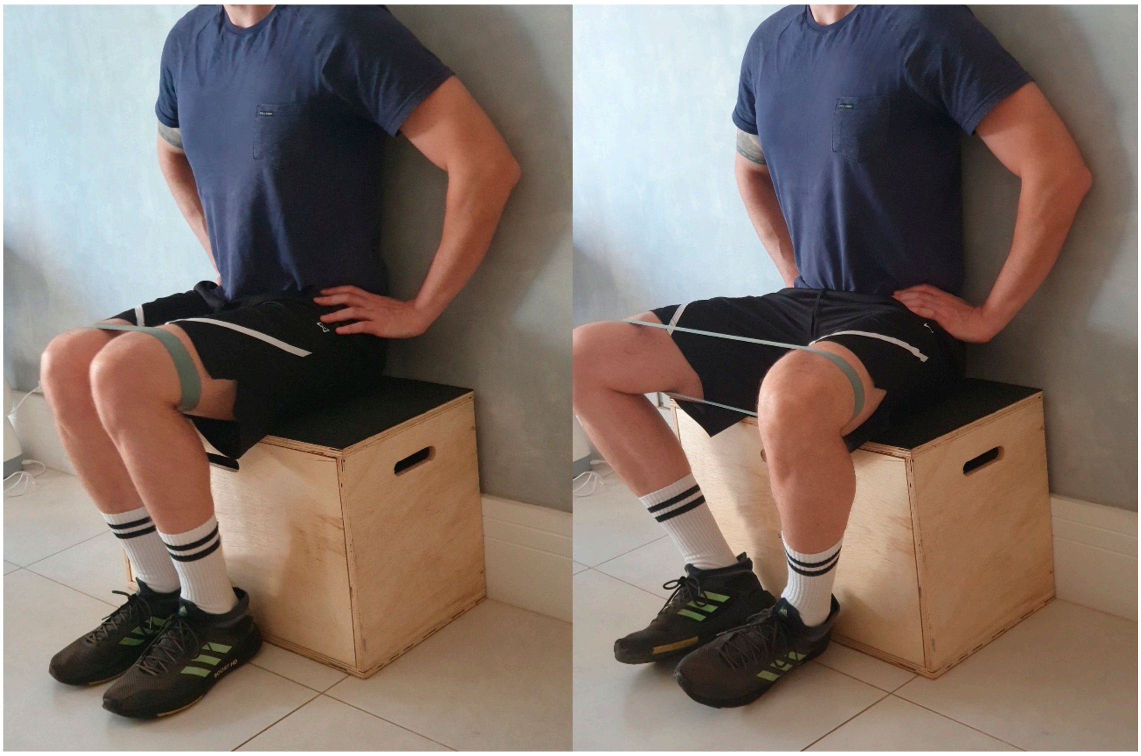

Supplement: Supplementary file 1 [file medsci-14-00312-s001.zip › medsci-4258775-Supplementary.pdf]
